# Iliopsoas Abscess, Spondylodiscitis, Septic Pulmonary Embolism, and Symmetrical Peripheral Gangrene by Hypermucoviscous Klebsiella pneumoniae

**DOI:** 10.7759/cureus.67985

**Published:** 2024-08-28

**Authors:** Hina Hamada, Koji Hayashi, Yukie Morikawa, Maho Hayashi, Akihisa Shirasaki, Chie Yamamoto

**Affiliations:** 1 Department of Internal Medicine, Fukui General Hospital, Fukui, JPN; 2 Department of Rehabilitation Medicine, Fukui General Hospital, Fukui, JPN

**Keywords:** septic pulmonary embolism, spondylodiscitis, iliopsoas abscess, symmetrical peripheral gangrene (spg), hypermucoviscous klebsiella pneumoniae, hypervirulent klebsiella pneumoniae, klebsiella pneumoniae invasive syndrome (kpis), klebsiella pneumoniae (kp)

## Abstract

Hypermucoviscous *Klebsiella pneumoniae* (hmKP) is a bacterium known to cause lesions, including abscesses, in various organs. Unlike typical *Klebsiella pneumoniae*, hmKP can yield a positive string test in culture specimens. Infections caused by hmKP often become severe and are associated with a poor prognosis. In this report, we describe a case of iliopsoas abscess, spondylodiscitis, septic pulmonary embolism, and symmetrical peripheral gangrene (SPG) caused by hmKP. The patient received non-invasive positive-pressure ventilation, hemodialysis, platelet transfusions, fresh frozen plasma, thrombomodulin, and intensive antibiotic therapy including rifampicin. The patient underwent 148 days of antibiotic therapy and achieved a good outcome.

## Introduction

Hypermucoviscous *Klebsiella pneumoniae* (hmKP), known as a hypervirulent type, was first reported in Taiwan in 1986 [1]. The first report describes seven cases of pyogenic liver abscess and septic endophthalmitis caused by *Klebsiella pneumoniae* (*K. pneumoniae*)* *[1]. After that, most of the reports came from East Asia, mainly Taiwan [2]. The clinical presentation was distinctive, as patients with no prior history of hepatobiliary disease developed community-acquired pyogenic liver abscesses and showed a tendency for hematogenous spread to distant locations [1,2]. In addition to the characteristic syndrome of liver abscess with hematogenous spread, it is known to cause extrahepatic infections including endophthalmitis, pneumonia, CNS disease (meningitis, brain abscess, and epidural abscess), urinary tract infections, biliary tract infections, severe skin, soft tissue and bone infections including necrotizing fasciitis, neck and psoas abscess, osteomyelitis, and bacterial peritonitis [2,3]. More recently, a high percentage of vascular thrombotic complications, including pulmonary embolus [4], has been reported, as well as one case of endocarditis [5]. The hmKP is distinguished from normal type by a positive string test [2]. The terms hypermucoviscous and hypervirulent are frequently used interchangeably in the literature; however, not all patients with a hypervirulent phenotype possess hypermucoviscous bacteria, and not all hypermucoviscous isolates result in an invasive syndrome [2].

In this report, we describe a case of iliopsoas abscess, spondylodiscitis, septic pulmonary embolism, and symmetrical peripheral gangrene (SPG) caused by hmKP with a favorable prognosis.

## Case presentation

An 82-year-old Japanese man developed back pain four days before admission. He had a medical history of diabetes mellitus, hypertension, alcoholic liver disease, cholecystolithiasis, chronic kidney disease, and carotid artery stenosis. He took several medications including trichlormethiazide, enalapril maleate, valsartan, cilnidipine, sitagliptin, allopurinol, ursodeoxychole, aspirin, loxoprofen, rebamipide, and clotiazepam. His family history was unremarkable. He took over-the-counter analgesics, but because his pain persisted he presented to a previous hospital for evaluation. During his evaluation he was found to be in disseminated intravascular coagulation (DIC) and the patient was transferred to our hospital. Vital signs on admission were a temperature of 36.4°C, blood pressure of 171/99 mmHg, pulse rate of 78 beats per minute, and oxygen saturation (SpO2) of 88% on room air. The patient was conscious and alert, there was no conjunctival pallor or jaundice noted on examination. Breath sounds were clear, with no adventitious sounds detected, and there was no asymmetry in lung sounds. The abdomen was flat and soft, with no tenderness, and bowel sounds were normal. The patient reported spontaneous pain in the left lumbar region. Cyanosis was noted in the distal extremities. Both dorsalis pedis arteries were palpable. Blood tests were significant for increased inflammatory response, anemia, thrombocytopenia, renal dysfunction, liver dysfunction, hyperglycemia, and coagulation abnormalities (Table 1).

**Table 1:** The result of blood tests on admission. PT, prothrombin time; PT-INR, prothrombin time - international normalized ratio; APTT, activated partial thromboplastin time; Fib, fibrinogen; FDP, fibrin degradation products.

Inspection items	Result	Reference range
White blood cell count	17500 /μl	(3300–8600)
Red blood cell count	405×10⁴ /μl	(386–492×10⁴)
Hemoglobin	11.9 g/dl	(11.6–33.4)
Blood platelet	2.0×10⁴ /μl	(15.8–34.8)
Total protein	6.1 g/dl	(6.6–8.1)
Albumin	2.2 g/dl	(4.1–5.1)
Alkaline phosphatase	632 U/l	(106–322)
Aspartate aminotransferase	95 U/l	(13–30)
Alanine aminotransferase	48 U/l	(7–30)
Lactate dehydrogenase	488 U/l	(124–222)
Creatine kinase	214 U/l	(41–153)
γ-glutamyltransferase	187 U/l	(13-64)
Total bilirubin	1.4 mg/dl	(0.4-1.2)
Direct bilirubin	0.9 mg/dl	(0-0.4)
Amylase	40 U/l	(44–132)
Blood urea nitrogen	84.4 mg/dl	(8.0–20.0)
Choline esterase	180 U/l	(240-486)
Uric acid	8.5 mg/dl	(3.7–7.0)
Creatinine	5.59 mg/dl	(0.46–0.79)
Natrium	136 mmol/l	(138–145)
Potassium	5.0 mmol/l	(3.6–4.8)
Chlorine	101 mmol/l	(101–108)
Glucose	267 mg/dl	(73–109)
Hemoglobin A1c	7.4%	(<5.5%)
C-reactive protein	>30.00 mg/dl	(0.00–0.14)
PT	16.1 sec	(9.6–13.1)
PT-INR	1.48	(0.8-1.2)
APTT	41.3 sec	(24-34)
Fib	550.6 mg/dl	(155-415)
FDP	>120.0 μg/ml	(<5.1)
D-dimer	54 μg/ml	(21.0–29.0)

**Figure 1:**
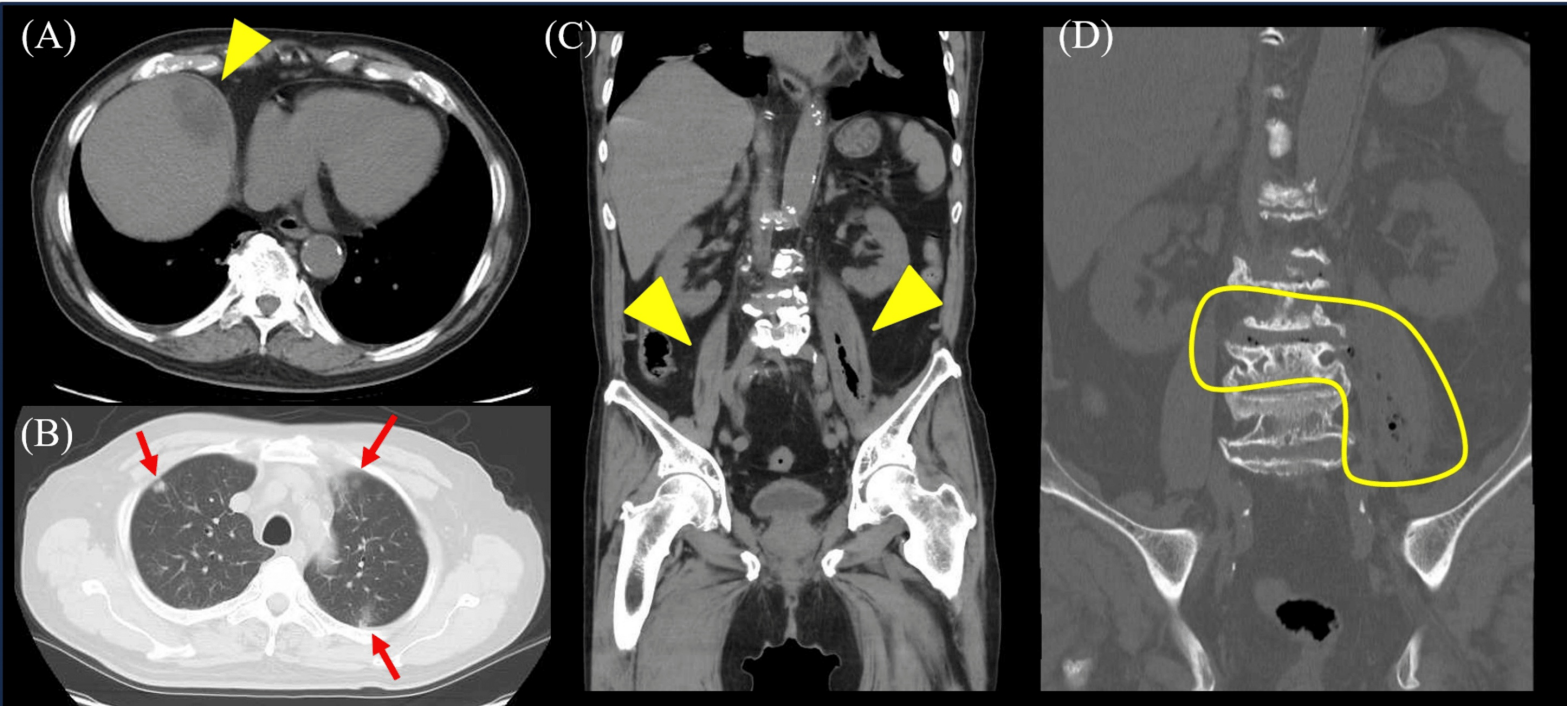
The result of thoracic and abdominal computed tomography (CT) on admission. (A) Abdominal CT showing a low-density area in the liver parenchyma which indicates a liver abscess (arrowhead). (B) Thoracic CT showing multiple nodular shadows suspected to be septic embolisms in the lungs (arrowheads). (C) Abdominal CT showing thickening of the bilateral, but predominantly left-sided, iliopsoas muscle with the presence of air within it (arrowhead). (D) Abdominal CT showing air within the left iliopsoas muscle and adjacent intervertebral disc.

Thoracic and abdominal computed tomography (CT) revealed a hepatic abscess (Figure 1A), bilateral iliopsoas abscess (Figure 1C-1D), spondylodiscitis (Figure 1D), and multiple nodular shadows suspected to be septic embolism in the lungs (Figure 1B).

**Figure 2:**
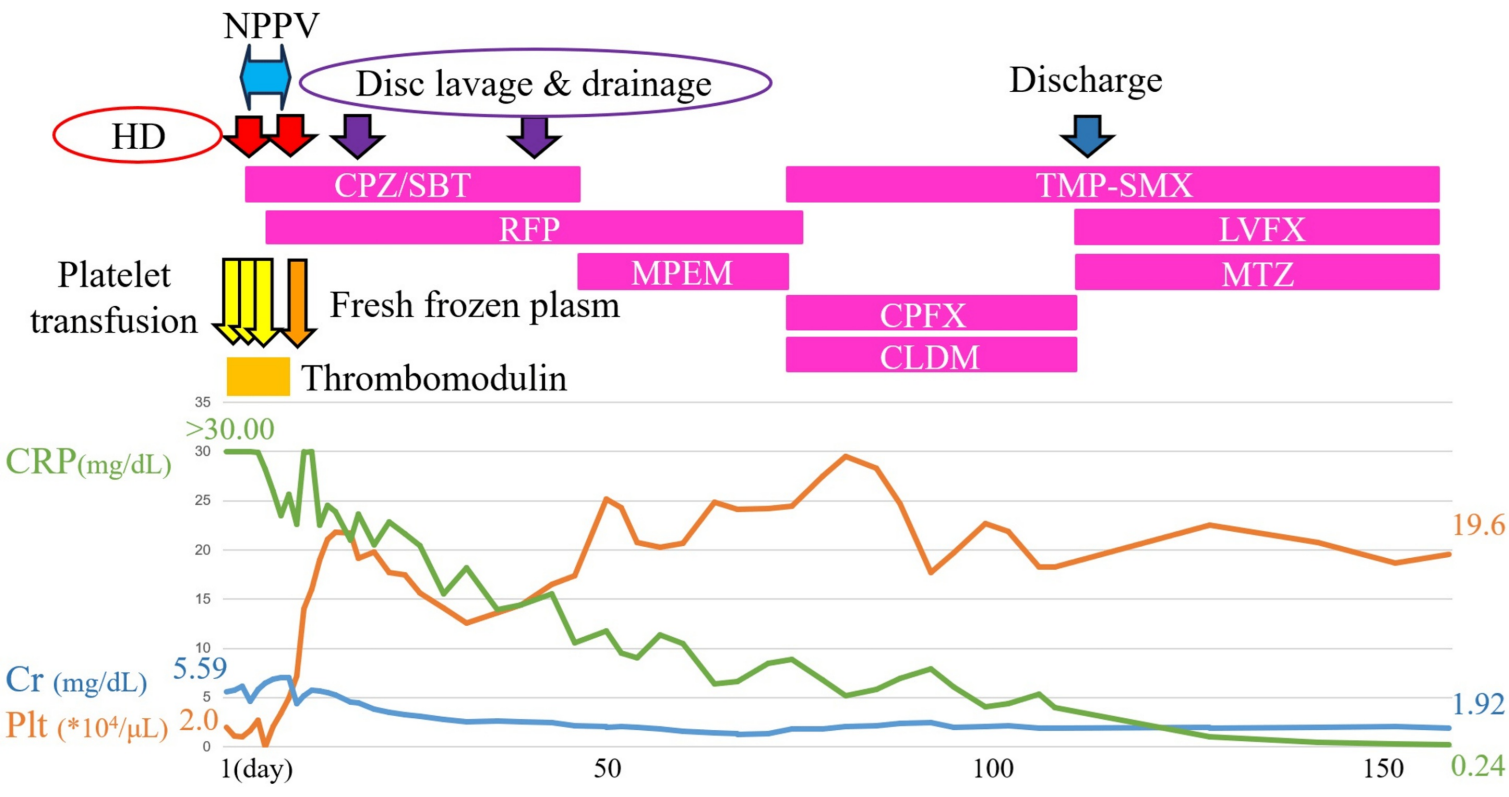
Diagram of time course about treatment and blood tests. The patient received hemodialysis (HD), platelet transfusions, fresh frozen plasma, thrombomodulin, non-invasive positive-pressure ventilation (NPPV), and antibiotic therapy. Upon admission, blood markers indicated severe thrombocytopenia, renal failure, and a heightened inflammatory response; however, these symptoms improved over time. NPPV, non-invasive positive-pressure ventilation; HD, hemodialysis; CRP, C-reactive protein; Cr, creatinine; Plt, platelet; CPZ/SBT, cefoperazone/sulbactam; RFP, rifampin; MEPM, meropenem; TMP-SMX, trimethoprim-sulfamethoxazole; CPFX, ciprofloxacin; CLDM, clindamycin; LVFX, levofloxacin; MTZ, metronidazole.

The course of the patient's illness after admission is shown in Figure 2. We started administering cefoperazone/sulbactam (CPZ/SBT) at a dose of 6 g/day, platelet transfusions, fresh frozen plasm, and thrombomodulin preparations, which are approved for the treatment of DIC in Japan, to treat the bacterial infection and DIC. Hemodialysis was initiated for severe renal failure. On day 3 of admission, two sets of blood cultures collected upon admission tested positive for a gram-negative rod, *K. pneumoniae*, and the string test also yielded a positive result. The antibacterial susceptibility test showed that the sensitivity to all evaluated antibiotics was favorable. Based on these results, we initiated treatment with rifampicin (RFP) at a dose of 450 mg/day in addition to CPZ/SBT.

**Figure 3:**
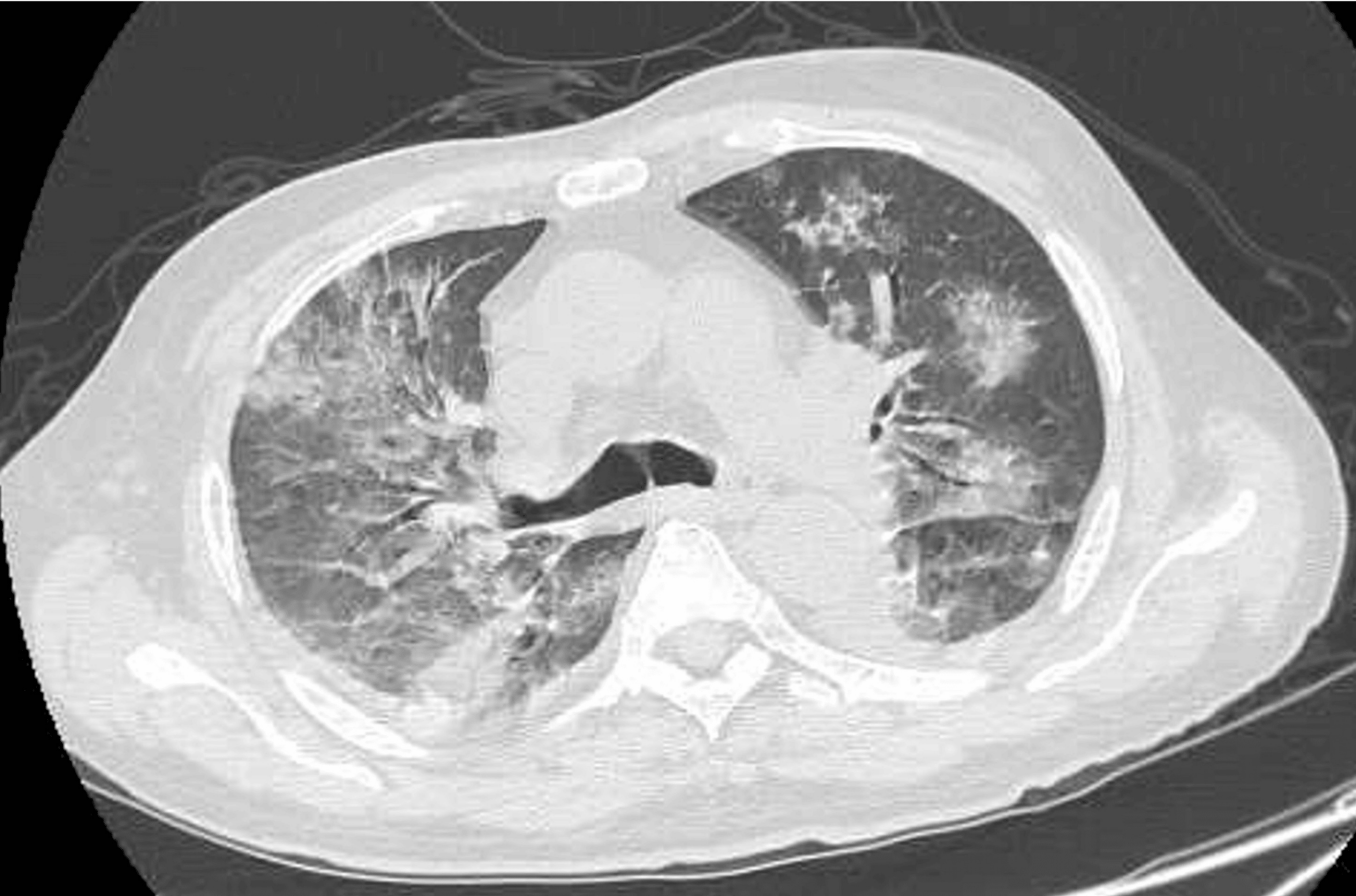
The result of thoracic CT on day 3. Thoracic CT showing a mix of widespread consolidation and ground-glass opacification in the lungs.

Repeat thoracic CT revealed that the infiltrates in both lungs worsened, and we diagnosed the patient with acute respiratory distress syndrome (Figure 3). Due to respiratory compromise, non-invasive positive pressure ventilation (NPPV) was initiated, the patient’s respiratory status stabilized, and he was weaned off on day 9. On day 16 when the platelet count recovered, disc lavage and drainage were performed, and a culture test of the disc aspirate on blood agar was positive for *K. pneumoniae*.

**Figure 4:**
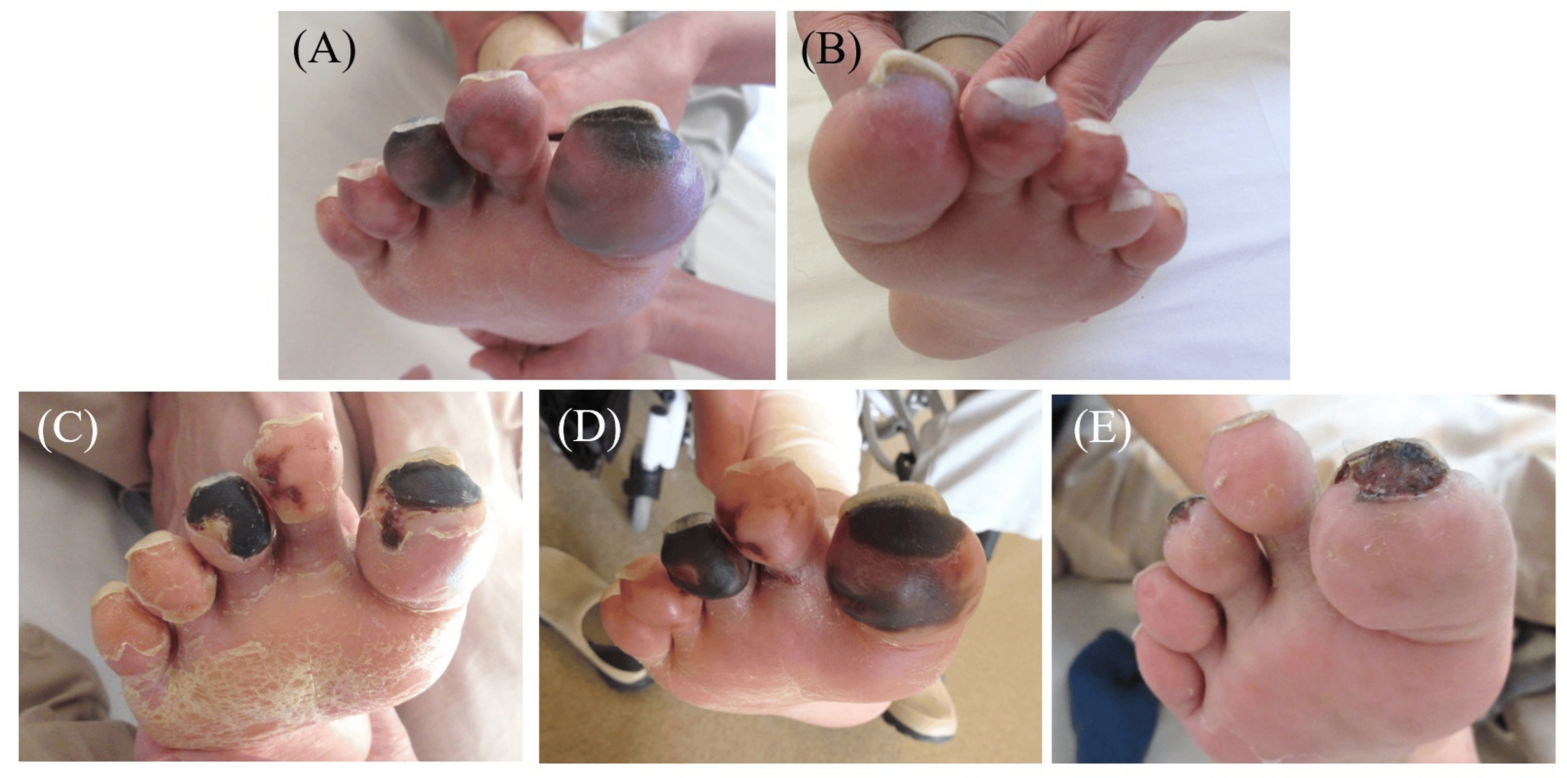
The photographs of the patient’s toes. (A, B) Photograph on day 17 showing that the tips of the patient's toes on both sides have turned a deep purple-to-black color. These findings led to a diagnosis of symmetrical peripheral gangrene (PSG) and prostaglandin therapy. (C) Photograph on day 31 showing the tips of the toes having turned black. Debridement was performed. (D) The photograph on day 45 showing the enlargement of the darkening lesion. Debridement was performed again. (E) The photograph on day 163 showing a remarkable reduction and improvement of the lesions on the toes.

Starting on day 17, cyanosis of the skin on the tips of both toes began to turn black, suggesting progression to SPG (Figure 4). We performed wound care after debridement. On day 42, C-reactive protein increased despite treatment with CPZ/SBT and RFP, so CPZ/SBT was switched to meropenem (MEPM) at a dose of 2000 mg/day and RFP 450 mg/day. On day 70, CT images confirmed the resolution of the liver abscess but showed persistence of the iliopsoas abscess, prompting a change in treatment from MEPM to a combination of ciprofloxacin (CPFX) 600 mg/day, clindamycin (CLDM) 1200 mg/day, and trimethoprim-sulfamethoxazole (TMP-SMX) at a dose of TMP: 320 mg/day, SMX: 1600 mg/day. By day 109, the iliopsoas abscess had completely resolved, and the antibiotics were switched to an oral combination of TMP-SMX, levofloxacin (LVFX) 250 mg/day, and metronidazole (MTZ) 1500 mg/day. The patient remained paralyzed in both lower limbs due to disuse syndrome but improved to the point where he was able to independently maneuver a wheelchair and was discharged on day 113. After confirming the resolution of the remaining iliopsoas abscess by CT, oral antibiotics were discontinued on day 148. On day 163, the patient was evaluated for SPG at the dermatology outpatient clinic, where a significant reduction and improvement in the toe lesions were noted (Figure 4).

## Discussion

In this report, we describe a case of iliopsoas abscess, spondylodiscitis, septic pulmonary embolism, and SPG caused by *K. pneumoniae*. Based on the result of a string test, *K. pneumoniae* was the hypermucoviscous type (hmKP). Based on previous reports, complications such as iliopsoas abscess, spondylodiscitis, and septic pulmonary embolism appear to be relatively common, whereas SPG is exceedingly rare.

SPG occurs in various medical conditions and is characterized by symmetrical gangrene in two or more extremities without large vessel obstruction or vasculitis [6]. The condition typically affects the fingers and toes, although it can occasionally involve the nose, earlobes, or genitals [6]. SPG may unpredictably manifest in cases of sepsis, low output states, vasospastic conditions, myeloproliferative disorders, or hyperviscosity syndrome. The condition has a high mortality rate, and survivors often require multiple limb amputations. DIC is commonly observed in SPG cases, and the consistent clinical presentation of SPG suggests that DIC may be the final common pathway in its pathogenesis [6]. To our knowledge, there is only one previous report of SPG associated with *K. pneumoniae* [7], but this case did not perform testing to confirm hmKP. Additionally, although SPG has a high reported mortality rate, with appropriate treatment, including antibiotics and debridement, the patient survived without requiring toe amputation.

Regarding antibiotics, we used several medications. The reason for initially choosing CPZ/SBT was the patient's renal dysfunction. The causative bacterium was identified as hmKP, and RFP was added during the acute phase. It is reported that the hypermucoviscous capsule is known to be a major virulence determinant and RFP elicits anti-mucoviscous activity against hmKP [8]. In addition, a case report has also demonstrated the efficacy of RFP against hmKP [9]. Ultimately, we transitioned to oral antibiotics, opting for a combination of three drugs, TMP-SMX, LVFX, and MTZ, because of the patient’s favorable response to this regimen. The patient underwent 148 days of intensive antibiotic therapy and achieved a good outcome, becoming somewhat independent in daily life, although he still uses a wheelchair. Long-term combination antibiotic therapy may be an effective treatment option for multiple abscesses throughout the body caused by hmKP.

## Conclusions

We report a case of iliopsoas abscess, spondylodiscitis, septic pulmonary embolism, and symmetrical peripheral gangrene (SPG) caused by hypermucoviscous *Klebsiella pneumoniae* (hmKP), successfully treated with long-term antibiotic therapy. Cases of SPG due to hmKP are extremely rare. Long-term combination antibiotic therapy may be an effective treatment option for multiple abscesses throughout the body caused by hmKP. Given the absence of reported cases of successful treatment for multi-organ abscesses with SPG caused by hmKP, this report is likely to be a valuable contribution to clinical practice.

## References

[1] Liu YC, Cheng DL, Lin CL (1986). Klebsiella pneumoniae liver abscess associated with septic endophthalmitis. Arch Intern Med.

[2] Choby JE, Howard-Anderson J, Weiss DS (2020). Hypervirulent Klebsiella pneumoniae - clinical and molecular perspectives. J Intern Med.

[3] Lee HC, Chuang YC, Yu WL (2006). Clinical implications of hypermucoviscosity phenotype in Klebsiella pneumoniae isolates: association with invasive syndrome in patients with community-acquired bacteraemia. J Intern Med.

[4] McCabe R, Lambert L, Frazee B (2010). Invasive Klebsiella pneumoniae infections, California, USA. Emerg Infect Dis.

[5] Rivero A, Gomez E, Alland D, Huang DB, Chiang T (2010). K2 serotype Klebsiella pneumoniae causing a liver abscess associated with infective endocarditis. J Clin Microbiol.

[6] Sharma BD, Kabra SR, Gupta B (2004). Symmetrical peripheral gangrene. Trop Doct.

[7] Yoo JH, Min JK, Kwon SS, Jeong CH, Shin WS (2004). Symmetrical peripheral gangrene complicating Klebsiella pneumoniae sepsis associated with antiphospholipid antibodies. Ann Rheum Dis.

[8] Tohda M, Oinuma KI, Sakiyama A (2023). Rifampicin exerts anti-mucoviscous activity against hypervirulent Klebsiella pneumoniae via binding to the RNA polymerase β subunit. J Glob Antimicrob Resist.

[9] Lin YC, Cao X, Mo YC, Xie CP, Zhang YF, Li N, Chen HL (2021). Successful treatment of hypervirulent Klebsiella pneumoniae bacteremia with combination carbapenem and rifampicin. IDCases.

